# Does gender matter? An analysis of men's and women's accounts of responding to symptoms of lung cancer

**DOI:** 10.1016/j.socscimed.2017.09.015

**Published:** 2017-10

**Authors:** Alice MacLean, Kate Hunt, Sarah Smith, Sally Wyke

**Affiliations:** a MRC/CSO Social and Public Health Sciences Unit, University of Glasgow, Top Floor, 200 Renfield Street, Glasgow, G2 3QB, United Kingdom; b Academic Primary Care, University of Aberdeen, West Block, Polwarth Building, Foresterhill, Aberdeen, AB25 2ZD, United Kingdom; c Institute of Health and Wellbeing, University of Glasgow, 25-28 Bute Gardens, Glasgow, G12 8RS, United Kingdom

**Keywords:** United Kingdom, Help-seeking, Gender comparative, Gender identities, Men and masculinities, Consulting, Lung cancer, Qualitative, Narrative analysis

## Abstract

Men are often portrayed - in research studies, ‘common-sense’ accounts and popular media - as reluctant users of health services. They are said to avoid going to the GP whenever possible, while women are portrayed, in presumed opposition, as consulting more readily, more frequently and with less serious complaints. Such stereotypes may inadvertently encourage doctors to pay greater heed to men's symptoms in ‘routine’ consultations. Although previous research has challenged this view with evidence, and suggested that links between gender identities and help-seeking are complex and fluid, gender comparative studies remain uncommon, and particularly few studies (either qualitative or quantitative) compare men and women with similar morbidity. We contribute here to gender comparative research on help-seeking by investigating men's and women's accounts of responding to symptoms later diagnosed as lung cancer. A secondary analysis of qualitative interviews with 27 men and 18 women attending Scottish cancer centres revealed striking similarities between men's and women's accounts. Participants were seen as negotiating a complex and delicate balance in constructing their moral integrity as, on the one hand, responsible service users who were conscious of the demands on health care professionals' time, and as patients who did not take undue risks with their health, in the context of an illness for which people are often held culpable, on the other. In accounting for their responses to symptoms, men and women drew equally on culturally-embedded moral frameworks of stoicism and responsible service use. Regardless of gender, the accounts portrayed participants as stoic in response to illness and responsible service users; and as people seeking explanations for bodily changes and taking appropriate and timely action. Our analysis challenges simplistic, 'common-sense' views of gendered help-seeking and highlights that both men and women need support to consult their doctor for investigation of significant or concerning bodily changes.

## Highlights

• Assumptions that help-seeking differs by gender abound and are rarely challenged.
• We undertook a gender comparative analysis of responses to lung cancer symptoms.
• Men's and women's accounts of responses to symptoms were strikingly similar.
• Culturally-embedded moral frameworks suffused accounts, regardless of gender.
• Accounts balance stoic and responsible service use vs appropriate and timely action.

## 1 Introduction

Galdas and colleagues note that “much of the empirical literature suggests that stereotypical (or “traditional”) gender roles and norms – culturally dominant behaviour considered to be essentially “masculine” and “feminine” – are an important factor that shapes *both* men's and women's health help-seeking behaviour” (Galdas et al., 2010, p19). Public narratives concerning men's help-seeking for illness have long reinforced beliefs that “rather than seek help, men will be strong, stoical and often silent in matters relating to health” (Robertson, 2003, p112). To some extent, this stereotype has been fuelled by well-known gender differences in use of primary care; analysis of routinely collected data on almost 3.8million patients in the UK, for example, shows higher mean number of visits to the general practitioner in females than males between the ages of 10 and 65 years (Wang et al., 2013). Furthermore, a qualitative synthesis of patients' help-seeking experiences and delays in cancer presentation identified men's “reluctance to seek help” (Smith, et al., 2005, p829) as a ‘third order construct’, concluding that “Men viewed help-seeking as not masculine enough … and indicated that women found help-seeking easier because of greater contact with health services for themselves and their family” (p828). Such statements reinforce a view that men's (under)use of health care is problematic, consulting for serious symptoms at a later stage, while women are presumed to consult more readily, frequently and with less serious complaints (Hunt et al., 2010) and perhaps by implication to be ‘over-users’ of health service resources. But such stereotypes, and the evidence on which they are based, are themselves problematic, as we argue below, and can have far-reaching implications, on men's and women's understandings of ‘gender appropriate’ consulting behaviours, on doctors' interpretations of symptoms according to gender (Arber et al., 2006; Lyratzopoulos et al., 2012, Schoenberg et al., 2003) and potentially on the ways researchers investigate, understand and draw conclusions from evidence relating to help-seeking for illness among men and women. The dearth of studies taking a gender-comparative approach to critically investigate whether or not the available evidence on men's and women's help-seeking bears witness to the public narratives has been identified as a clear weakness in the evidence base (Hunt et al., 2010). In particular, Hunt and colleagues argue that “more critical gender-comparative research is needed to understand the ways in which men and women's help-seeking is similar or different to avoid medical bias in consultations (based on false premises about readiness to consult) and to develop gender-sensitive policy and practice on the most appropriate use of health services” (p253). Similarly, Galdas et al. (2010) argue for a need to go “beyond the masculine-feminine binary” of stereotypical gendered constructions of “stoic men” and “vulnerable or accommodating women”.

## 2 Gender identities and help-seeking for illness

In Western cultures, hegemonic masculinity, that is the idealised practices of masculinity to which men are thought to aspire, emphasises stoicism, independence, emotional control and a strong, healthy body (Connell, 1995). The concept of hegemonic masculinity is related to narratives of help-seeking and help-seeking behaviour because, within many current constructions of hegemonic masculinity, acknowledging illness and asking for help are viewed as signs of weakness and men are believed to be less likely than women to seek help when ill so as to avoid jeopardising performances of hegemonic masculinity (e.g. Addis and Mahalik, 2003, O'Brien et al., 2005, Robertson, 2006). Courtenay (2000, p1389) argued that health-related beliefs and behaviours are a means of demonstrating gender, and that dismissing health concerns is a key practice of hegemonic masculinity:

“By dismissing their health care needs, men are constructing gender. When a man brags, ‘I haven't been to a doctor in years', he is simultaneously describing a health practice and situating himself in a masculine arena.”

Links between health-related behaviours and performances of masculinity are commonly asserted to: lead to men making less (or inappropriately late) use of health-care services than women; have a detrimental impact on men's morbidity and mortality; and explain, at least partly, men's shorter life expectancy compared with women (Baker, 2016, Banks and Baker, 2013, White, 2011). In contrast, it has been argued that “feminine ‘ideals’ (in the context of help-seeking behaviour) are typically seen as asking for help, caring about health, nurturing and monitoring partners' and children's health and well being” (Galdas et al., 2010) (p18), linking stereotypes of women's greater ‘propensity’ to visit the GP to their presumed caring roles.

While there is evidence of men presenting themselves as avoiding seeking help for various reasons, including the perceived need to present themselves as traditionally masculine (see Galdas et al., 2005 for examples), research reports that the links between masculinities and help-seeking are not straight-forward. Robertson, 2003, Robertson, 2006 argued that men are caught in a dilemma between ‘don't care’ and ‘should care’, and feel they need to legitimise their health service use to avoid potential emasculation. Others have shown how expressions of masculinity in relation to help-seeking, and the extent to which men justify health service use, can vary by ethnicity, culture (Galdas et al., 2007), age, occupation and medical history (O'Brien et al., 2005). Indeed, others have suggested that help-seeking can be reformulated by (at least some) men as a masculine act which signifies taking control and responsibility to solve health problems (Farrimond, 2011, Johnson et al., 2012). Such research highlights the importance of destabilising assumptions that hegemonic masculinity precludes help-seeking for all men in all contexts or that help-seeking is always disruptive to hegemonic masculinity. More research is thus needed to investigate the complex and fluid links between gender identities and help-seeking (see also, Galdas et al., 2010).

## 3 How best can we investigate the links between gender and help-seeking for illness?

A synthesis of research on gender and access to healthcare reported a dearth in comparisons of men's and women's responses to the same health concerns, concluding that “[a] full and comprehensive answer to the question of whether access to healthcare is characterised by gendered patterns of advantage and disadvantage is thus not possible” (Annandale et al., 2007, p477). Hunt et al. (2010) called for two types of gender comparative approaches to address this gap: quantitative studies comparing patterns of help-seeking behaviour amongst men and women with similar underlying morbidity; and qualitative studies critically investigating and comparing men's and women's accounts of their decisions to consult (or not) when they experience symptoms.

Recent quantitative studies examining GP consulting rates among men and women with comparable morbidity challenge stereotypes of stark gender differences in help-seeking behaviours. A systematic review of consultation for headache and back pain found surprisingly weak evidence of greater consultation amongst women (Hunt et al., 2011). Analyses of a large routinely collected primary care data source revealed small gender differences in men's and women's rates of consulting when accounting for underlying morbidity, such as depression and cardiovascular disease (Wang et al., 2013), and that, in relation to three non-sex specific cancers (colorectal, lung and malignant melanoma), there is little evidence that men are being diagnosed at a later stage than women and very little evidence of gender differences in GP consulting in the 24 months prior to diagnosis (Wang et al., 2014). The authors assert that their analyses provide little support for the hypothesis that gender differences in mortality might be explained by men presenting later or less to primary care. A study which examined the number of GP consultations prior to hospital referral for cancer found the probability of making three or more pre-referral consultations was greater for women than men (Lyratzopoulos et al., 2012), and particularly marked for bladder cancer. The authors highlight an apparent danger of GPs “misattributing urinary tract symptoms in women to a benign cause” (p363) and suggest GPs' readiness to suspect cancer varies by cancer type and gender, meaning that, for some cancers, women may have to make more visits to their GP before cancer is considered as the possible cause of symptoms.

Findings from qualitative gender comparative research also challenge gender stereotypes. A study of self-management of multiple morbidity in mid-life revealed that men and women accounted for the management of illness within a moral framework, presenting their use of healthcare as a last resort and demonstrating an obligation to manage symptoms ‘well’ (Townsend et al., 2006, Townsend et al., 2008). Galdas et al. (2010), interviewing men and women diagnosed with an acute coronary syndrome, found that the help-seeking behaviours described did not always align with stereotypical masculine and feminine ideals. Some women narrated their reactions during a cardiac event by emphasising stoicism, control and endurance, whereas many of the responses that men described entailed explicit concern for their health, relinquishing control to health professionals and displays of vulnerability. These studies demonstrate the importance of conducting systematic explorations of the personal accounts of help-seeking for illness to investigate whether, and in what ways, they substantiate or contradict prevalent narratives of men's and women's consulting behaviours.

## 4 A gender comparative exploration of accounts of experiencing symptoms later diagnosed as lung cancer

In the UK, lung cancer (LC) is the second most common cancer, accounting for 13% of all new cancer cases. In 2013, around 45,500 people were diagnosed with LC and in 2014 just under 36,000 people died from it (Cancer Research UK, 2016). Cancer Research UK (2016) reports that “when diagnosed at its earliest stage, more than a third of people with lung cancer will survive their disease for five years or more, compared with around 5 in 100 of people when diagnosed at a later stage”. It has been suggested that the period before seeking help for symptoms is when the greatest delay in LC diagnosis occurs (Corner et al., 2005). Proposed reasons for this include symptom normalisation (Brindle et al., 2012, Corner et al., 2006) and the role of social factors, such as smoking-related stigma (Chatwin and Sanders, 2013). Therefore, finding ways to promote earlier diagnosis is an important focus of UK LC research (Smith et al., 2013).

Research which takes a gender comparative approach to help-seeking for cancer is sparse but reveals mixed results. A review of barriers and facilitators to engagement in symptom reporting and screening found that lack of awareness of LC symptoms is more prominent amongst UK men than women (Braybrook et al., 2011). Quantitative assessments of the time taken to consult with symptoms of LC show no significant differences between men and women (Smith et al., 2009, Wang et al., 2014). Additionally, a systematic review of patient-mediated delay in consulting for common cancers revealed “little evidence of any association between sex and time to presentation […] for lung cancer” (Macleod et al., 2009, pS93). Birring and Peake (2005) found that over half of the patients in their study, both men and women, needed to be encouraged by family and friends before consulting their GP. However, Bowen and Rayner (2003) reported that men delayed consulting longer than women. A metasynthesis of qualitative studies of help-seeking and delay in cancer presentation highlighted that men reported help-seeking for illness is not masculine and should only be done in extreme cases, while women reported prioritising family health and domestic demands above their own health concerns (Smith et al., 2005).

Although cancer generally attracts more stigma and fear than other illnesses (Cancer Research UK, 2011), stigma is greater in relation to LC than other cancers (Chambers et al., 2012, Marlow et al., 2015) and experienced more by LC patients who have smoked than non-smokers (LoConte et al., 2008). This may be due to perceptions associated with LC, such as a poor prognosis and the prospect of a horrible death, mass media campaigns using graphic and shocking images to persuade smokers to stop and the known link of LC with smoking, often leading to understandings of LC as self-inflicted (Chambers et al., 2012, Marlow et al., 2015). Even LC patients who have never smoked report the assumption, by health professionals and lay people, that smoking caused their illness, while some who have smoked feel delays in diagnosis can be caused by GPs not taking a ‘smoker's cough’ seriously (Chapple et al., 2004). Thus, the moral discourses around causation of LC and the stigma this brings may result in reluctance to present to health services and contribute to perceptions that seeking treatment will be futile (Chapple et al., 2004, Corner et al., 2006).

In this paper, we present results of a secondary analysis of men's and women's accounts of the lead-up to consulting their GP with symptoms later diagnosed as LC. The paper aims to add to existing qualitative gender comparative research by furthering understanding of the links between gender identities and accounts of help-seeking for comparable morbidity. Our analyses centre on the exploration of the moral aspects of men's and women's accounts of the onset of bodily changes and their subsequent interpretations and actions.

## 5 Methods

The data presented in this paper derive from a mixed methods study which involved a quantitative survey, review of hospital and GP case notes, and qualitative interviews (Smith, 2010; Smith et al., 2009). Ethical approval for the primary study was obtained from the North of Scotland Research Ethics Committee and North Glasgow University Hospitals Trust Research Ethics Committee. Patients who had recently been diagnosed with LC were invited to take part in a face-to-face quantitative survey by specialist nurses at two Scottish cancer centres. Agreeable patients were given information sheets and asked if they would allow their contact details to be passed to the research team. Patients (n = 306) who completed the quantitative survey were then asked for permission to be contacted again to discuss taking part in a semi-structured interview. Sixty-eight per cent (n = 244) were willing to be contacted again by telephone either as soon as possible after the quantitative interview (usually within one week) or at a point specified by the participant. Purposive sampling was used to ensure we included people with a range of socio-demographic characteristics that might influence a participant's response to lung cancer symptoms, such as the relative deprivation of the area in which they lived, age and gender (Ritchie, et al., 2003), and allow comparison of the accounts of those who consulted within twelve weeks with those who consulted later.

### 5.1 Qualitative interviews

Qualitative interviews were conducted with 45 participants shortly after diagnosis (38 in participants' homes, 6 in hospitals, and one by telephone). Morse (2002, p317) describes the narrative account of an illness as having a distinctive beginning, middle and end with the person conveying their account as “one thread, weaving all of the associated events that are relevant into a sequential tale”. To facilitate participants’ construction of events, from first bodily change(s) to diagnosis, a narrative approach was used. In line with Elliot (2005), who argues that the best questions for narrative interviews invite participants to talk about specific times and situations, the researcher in this study (SS) began each interview saying:

“In this interview we would like you to tell us what happened from the time you first thought there might be a problem until you were referred to the hospital. So thinking back to before you went to your GP (or other medical help) tell me why you first thought there might be a problem?”

There was minimal interjection from the researcher while participants constructed their narrative, except for encouraging non-verbal communication or utterances. A semi-structured topic guide was then used to explore topics that did not arise spontaneously, such as relationships with GPs and perceptions of LC causes and risks. Similar narrative approaches have been used in numerous other qualitative studies (Reissman, 2002), including those with lung cancer patients (Chapple et al., 2004, Levealahti et al., 2007). Interviews were recorded with the participants’ consent and transcribed verbatim. Most interviews (n = 36) ranged between 40 and 90 min long, six were 90 min to two hours and three lasted approximately 30 min.

Table 1 provides a summary of participant characteristics. Twenty-seven men and 18 women were interviewed, reflecting the gender balance of the quantitative sample, which in turn reflected gender patterning in the incidence of lung cancer generally. Most were aged between 50 and 79. There was a roughly even split between participants who had consulted their GP within 12 weeks (n = 25, 16 male) and those who waited longer (n = 20, 12 male), and diversity by socio-economic and smoking status. Most lived in urban areas and all were White, reflecting the relative lack of ethnic diversity in these areas.

**Table 1:** Participant characteristics.

Participant Identity number	Sex	Age	Time to consultation (Early <12 weeks; Late > 12 weeks)
1	M	60–69	Late
6	M	70–79	Late
7	F	50–59	Early
12	M	50–59	Late
20	M	60–69	Late
37	M	>80	Late
41	M	60–69	Early
43	M	70–79	Late
53	F	70–79	Late
94	M	50–59	Early
98	F	60–69	Early
119	F	>80	Early
123	M	<49	Late
135	F	>80	Late
138	F	50–59	Late
151	M	>80	Early
155	M	50–59	Early
163	F	60–69	Late
164	F	50–59	Early
167	M	60–69	Late
183	F	<49	Early
500	M	70–79	Early
502	M	50–59	Early
508	F	50–59	Late
521	F	60–69	Late
524	M	60–69	Early
529	F	70–79	Early
563	F	70–79	Late
569	M	<49	Early
577	F	<49	Early
582	F	60–69	Early
584	M	50–59	Early
611	M	70–79	Early
612	M	60–69	Late
619	M	50–59	Early
622	M	50–59	Late
629	M	50–59	Late
630	M	70–79	Early
638	M	60–69	Late
639	F	70–79	Late
641	F	70–79	Early
642	M	60–69	Early
650	F	50–59	Early
653	M	60–69	Early
655	M	60–69	Early

### 5.2 Analysis

We conducted secondary analysis, undertaking a new exploration of the qualitative data to address a different research question. While some believe that only researchers who gathered the data can best conduct analysis, others argue that secondary analysis can benefit from the ‘distance’ that a new researcher has from the data (Ziebland and Hunt, 2014). However, involvement of the primary researchers in providing contextual details of the study and data collection, as well as contributing to interpretations of the data, is key to secondary analysis (Emslie et al., 2007).

The first stage of analysis involved familiarisation, with AM reading all transcripts numerous times and annotating transcripts with early analytical thoughts, such as noting potential themes and highlighting language. AM discussed the emerging findings with two co-authors, SW and KH. The next stage entailed a structured, thematic approach (Ziebland and McPherson, 2006), whereby AM developed an initial coding frame based on broad emergent themes which was refined through discussions with SW and KH who had each read, independently, 10 transcripts. Nvivo 10 was used by AM to facilitate systematic application of the broad coding frame to all transcripts and aid retrieval of data. The coding frame at this point included four broad themes relevant to this paper (‘symptoms’, ‘time/actions’, ‘moral self’ and ‘experiences of health services’). Three co-authors (AM, SW, KH) read the content of these codes, paying attention to how participants recounted experiences of bodily changes, what they said about how they interpreted, evaluated and reacted to these and how they presented themselves within this story. Guided by Radley and Billig's (1996, p231) approach, we were interested in understanding participants' narratives and words in terms of what they were “rhetorically accomplishing” rather than as simply representing a ‘true’ account of events. At this point, AM used a ‘Framework’ approach (Ritchie et al., 2003) within NVIVO, enabling comparisons and the identification of patterns and irregularities across each code. Participants' accounts were analysed without reference to gender before codes were systematically compared by gender.

Others have outlined ways in which, when narrating illness management, men and women construct their “moral worth” in various ways (Townsend et al., 2006). Similarly, our analyses revealed two over-arching themes which entailed dual notions of ‘responsibility’ (‘presentations of self as stoic in response to symptoms and responsible health service users’ and ‘presentations of making sense of symptoms and responding in appropriate, responsible and timely ways’) and which demonstrated the impact of moral frameworks on participants' presentations of their symptoms, interpretations, and subsequent actions. Through their accounts men and women appeared to interweave these dual constructions of behaving ‘responsibly’ to reconstruct their moral integrity as responsible health service users who did not take undue risks with their health in the face of a diagnosis for an illness for which people are often held culpable, as explored below.

## 6 Findings

Our analyses revealed more similarities than differences in how men and women talked about the lead-up to going to the GP. Participants described a range of experiences in relation to bodily changes. Some spoke of an absence of changes, some described experiencing changes over a considerable period of time (sometimes years), some talked of gradual worsening and accumulation of changes, while others described one stand-out change (such as the discovery of a lump). Therefore, similar to existing research (Levealahti et al., 2007), participants frequently portrayed the onset of LC, and recognition of bodily changes as a problem, or as possible symptoms of an illness, as a gradual, often lengthy and complex process.

In the sections below, we provide evidence of the ways in which men and women reconstructed their moral integrity through their accounts. Firstly, we show how participants presented themselves as stoic in response to bodily changes, often by emphasising how debilitating these had become before they had sought help, and as responsible health service users, by presenting themselves as only using overstretched GP services when absolutely necessary. Secondly, we demonstrate how they accounted for their efforts to make sense of bodily changes and portrayed themselves as having taken sensible, appropriate and timely actions. Within this second section, we highlight the ways in which participants' accounts differed according to the changes or symptoms they described. While we acknowledge that coming to understand various bodily changes as possible symptoms of an illness is often a lengthy process and those experiencing such changes rarely interpret them as symptoms immediately, to avoid ambiguity we will use the word ‘symptom(s)’ from here on to refer to the various bodily changes which participants described. Demographic information is given for all quotations below, including: participant identity number (followed by M (man) or W (woman)); age; and whether they consulted within or after 12 weeks (early/late). Use of the terms ‘early’ and ‘late’ is based on previous research which defines waiting longer than 12 weeks to consult as ‘late presentation’ due to adverse impact on survival rates (Richards et al., 1999). All quotes have been changed from local dialect to standard English.

### 6.1 Stoic in response to symptoms and responsible health service users

Participants reflected on the ways in which they generally used health services, using these accounts to build a picture of their moral identities and cultural values (Townsend et al., 2014). At the same time as they appeared eager to avoid being seen as having gone to the doctor unnecessarily, implying that doing so represents ‘irresponsible’ or wanton use of already overstretched services, they also appeared keen to present themselves as having reacted to their symptoms appropriately and responsibly. On the whole, men *and* women presented themselves as seldom going to the GP and not being “one for running to the doctor”:

“if I feel healthy enough I don't go [to the GP] you know I've got to be really down before […] I'll go […] I'm not one for you know running to the doctors and running off on the sick […] I just you know get on with it.” (653M, 60–69, early).

Across the participants' accounts there were a number of justifications for this way of using GP services. Some presented themselves as reacting to symptoms with stoicism, suggesting that it is in their ‘nature’ to only consult the GP as a last resort. Other participants talked about not wanting to waste their doctor's time unnecessarily or to prevent other, perhaps more ‘deserving’, patients from being seen, and they also distanced themselves from ‘malingerers’ who are “never away from the doctor's” (584M, 50–59, early), thus presenting themselves as aware that GP services are under pressure and should only be used when absolutely necessary. Other participants said they had not consulted very often simply because they were generally fit and healthy. As proof of infrequent consulting, both men and women referred to their thin medical record and practice staff comments that they did not see them often.

“I don't lie down to any kind of illness […] See I'm not a doctory person, I'm not a kind of person for, if you got my medical records and you looked at them, you'd see I'm not a person for going backwards and forwards eh, I mean I did go after when I knew there was something wrong […] I don't go to the doctor's for the least wee complaint or anything like that. Because that's what my doctor […] says to me, ‘It's so funny lookin’ at your records,’ he says ‘It's all blank, blank, blank, blank, blank,’ he says ‘and then all of a sudden, you've got all this, this test, that test, results for this, results for that’” (508W, 50–59, late).

Both men *and* women also talked about being urged to go to the GP by spouses, family members, friends, or colleagues (see also Molassiotis, 2009, Townsend et al., 2014), something frequently portrayed as defining masculine help-seeking behaviours. Indeed, the overall similarities in how men and women presented themselves as responsible and thereby (at least to some degree) reluctant, or certainly discerning, health service users contradict public narratives which depict simple binaries of men as reluctant and women as willing to seek medical help (Galdas et al., 2010, Townsend et al., 2014).

The onset of illness can be narrated as a biographical as well as diagnostic process (Levealahti et al., 2007). In presenting their attitudes towards, and histories of, health service use as aspects of their wider biographies, and indeed moral identities, participants in this study could be seen as positioning themselves to deflect any potential blame which their accounts of their LC symptoms and subsequent reactions may incur. In other words, if enduring illness without seeking medical advice is integral to a sense of self then they are not to be blamed for trying to cope with their symptoms without help, nor any consequent delay in diagnosis. Tensions were sometimes evident in accounts, perhaps due to the fact that two culturally valued behaviours, managing symptoms without using scarce GP resources and seeking help promptly for a serious illness, were being balanced precariously against one another, reflecting other research which highlights patients' dilemmas and the “moral question of cautious healthcare use” (Llanwerne, Newbould, Burt, Campbell & Roland, 2017). This was the case when some participants suggested that their tendency not to consult the GP had resulted in them delaying help-seeking for longer than they ‘should’ have. One man said “it never really entered my head to go to the doctors […] in hindsight I should've maybe went […] six months before” (622M, 50–59, late). Similarly, a woman said “I should have been at the doctor but I never was one to run to the doctor and just put it off” (135 W, >80, late).

### 6.2 Making sense of symptoms and responding in appropriate, responsible and timely ways

All participants, men and women, gave an account of the cognition work they had engaged in to make sense of, or try to explain, their symptoms and appeared keen to present themselves as having reacted appropriately, responsibly and in a timely fashion, therefore showing how they had not deliberately ignored symptoms of a potentially life-threatening illness. Given the moral frameworks that may have been influencing participants' accounts, it is possible that they felt the need to justify their reactions to their symptoms, regardless of whether this was because they delayed seeking help or sought help promptly. Men's and women's accounts of their cognition work and subsequent responses varied according to the symptoms they described.

Some men and women described themselves as shocked by their diagnosis, often linking this to an absence of symptoms, the trivial nature of symptoms, the lack of disruption to their everyday lives, or a sudden onset of symptoms with no prior signs of illness:

“If I hadn't been told I've got lung cancer, I wouldn't know. I have no pain, no symptoms, the only thing is I'm still kind of short of breath at times” (582W, 60–69, early)

“When you hear of people […] getting [cancer], they're in pain or there's something wrong with them […] but I could do everything […] I could walk up, down […] I just couldn't understand it.” (611M, 70–79, early)

Among these participants, men and women claimed to have “no warning”, “nothing obvious” or “no way I would have known”, thus implying that they could not have acted any differently based on their bodily experiences. Closer examination of these accounts revealed how, despite describing themselves as “fine” and their symptoms as “little things”, men and women often described experiencing a considerable number of symptoms. However, it was perhaps because they said they were “still managing” that they tended to trivialize their experiences:

“I didn't associate it really with being so unwell [the wheeze] was only when I lay down at night […] it didn't bother me through the day […] it wasn't affecting my health, and it wasn't making me not sleep […] my appetite wasn't affected […] it was more of annoyance than anything.” (650W, 50–59, early)

Other participants described their awareness of symptoms but highlighted that these had not fitted with their understandings of LC, or cancer more generally. One woman said she was “never short of breath, never wheezy” (138 W, 50–59, late) and one man said “I've never had any symptoms where I would have said, ‘Oh that's lung cancer’” (6M, 70–79, late). Participants commonly referred to a lack of pain as another reason for not suspecting cancer. On one level, these findings represent an apparent lack of knowledge among men and women as to the different ways in which LC can become manifest. It is also possible, however, that participants' portrayals of themselves as having “no warning” of LC is further evidence of the influence of moral frameworks; by showing that they did not ignore obvious signs of LC they were perhaps attempting to mitigate themselves from any blame which their consequent actions (or lack of) may incur. Indeed, one woman expressed anger that her medical notes listed symptoms which she felt were well known to be ‘lung cancer symptoms’, suggesting she felt it impugned her good judgment and status as a person who takes care of her health by consulting appropriately:

“I didn't feel any side effects that there could be something wrong with me as far as my lungs went […] [Consultant] said ‘I see here that the reason you're here is because you've got a dirty spit and a loss of weight?’ I said ‘That's not true and I want that taken off that! […] [I felt] very angry. […] Loss of weight is a bad thing if you're not dieting […] you worry right away […] and the dirty spit is not the thing you'd be happy about […] and that didn't happen, no way!” (641W, 70–79, early)

As previous research has found (Corner et al., 2006, Molassiotis, 2009; Smith et al., 2005), men and women who described symptoms which were easy to give ‘common sense’ explanations to, or worsened gradually, highlighted their normalisation of them by attributing them to: common minor illnesses; existing diagnoses; circumstantial explanations (e.g. stress); lifestyle factors (e.g. smoking); injuries; and ageing. For example:

“I mean a sore head's a sore head but what I felt was […] an ache rather than a pain […] then it went onto my shoulder […] actually to be quite honest I [was] putting it down as a sciatica […] cause once you reach my age then anything goes” (151M, >80, early)

Men's and women's accounts were peppered with words and phrases such as “honestly” and “to be quite honest”, suggesting a perceived need to justify their symptom assessments and convey that they did not (at least initially) suspect a serious illness. Indeed, many specifically stated that cancer never occurred to them, with striking similarities, across and within men's and women's accounts, in the language used. One man, who thought he had a “heavy cold”, added “I never in my world dreamt what I had, honestly” (611M, 70–79, early) and a woman, who attributed her symptoms to grief and stress, said “[I] never dreamt [I had lung cancer, it] never even entered my head” (563 W, 70–79, late). Although most participants tried to show that they could have taken no other course of action based on their symptoms, an undercurrent of self-blame was evident for a small number of men and women who admonished themselves for not being more questioning of their lingering and/or worsening symptoms. One man, who experienced a persistent cough for over six months, said “I should have thought, ‘Why am I coughing?’” (622M, 50–59, late).

Other participants described what could be considered more typical ‘lung cancer symptoms’ (e.g. haemoptysis, a lump or swelling, rapid or unexplained weight loss and severe pain). Some, again men and women, spoke of how they had initially found normalising explanations. One woman described how, on finding a lump on her neck, she (and her GP) thought it was swollen glands and she waited three months before consulting again because “it wasn't sore or annoying […] it wasn't easing down [but I] couldn't say I thought it was serious” (521 W, 60–69, late). Similarly, a man spoke of how he rationalised his haemoptysis, saying that due to “the racking of the chest with the coughing, I thought I'd burst a wee blood vessel” (630 m, 70–79, early).

Others also described ascribing ‘common sense’ explanations, albeit acknowledging that the possibility of cancer had crossed their minds. One woman recalled how, after coughing up blood two mornings in a row, she thought “It has got to be one of two things; either a really nasty chest infection or that [LC]” (163 W, 60–69, late). When narrating his rapid weight loss, one man referred to knowing others with LC who had lost weight, making him think “there could be a serious problem” (653M, 60–69, early). Nevertheless, while saying cancer was on their minds, some participants claimed they “didn't start panicking” (577 W, 40–49, early) and one did not share his suspicions with his GP in case he would be seen as paranoid. It is significant that participants were careful to highlight that they had not immediately interpreted their symptoms as caused by cancer, and possible that this was to avoid being seen as irrational or over-reacting and part of the balancing act which giving their accounts appeared to involve.

In general, however, participants who experienced symptoms more commonly associated with cancer spoke of them as triggers to seeking help promptly (within twelve weeks) without justifying this a great deal:

“I'd felt a lump in my neck, I think this was the Friday. And first thing on the Monday morning I went to the doctor” (577W, 40–49, early)

“We had a cough about seven weeks but we had a anti-biotic eh which didn't seem to do nothing then we got another tablet […] before we finally went down about the cough ehm the, the breathlessness, slightest bit of exertion and we was huffing and puffing. We knew then besides that and taking [coughing] up a wee bit of blood that there was something amiss.” (155M, 50–59, early).

Again, this demonstrates the ways in which some symptoms were presented, by men and women, as triggering immediate action because they were more recognisable as ‘lung cancer symptoms’. This could also be seen as part of participants' reconstruction of their moral integrity because presenting themselves as acting in appropriate, responsible and timely ways involves showing how they responded promptly to symptoms which were more recognisable or typical of LC and cancer more generally.

However, there were two men and one woman who had strongly suspected that they had LC yet said they delayed consulting their GP. All three described themselves as in severe pain and two had lost weight. All three were smokers when diagnosed and, although the woman said she did not believe this placed her at increased risk, both men acknowledged that as smokers they were at greater risk of developing LC. One of the men was a health professional, presumably with good knowledge of LC symptoms, while the woman had recently watched her father die of LC, perhaps implying increased knowledge of LC symptoms, and she perceived herself to be at increased risk due to the family history of LC. Both men voiced opinions about help-seeking for LC being futile. One said “it didn't matter how quick I went to see them [health professionals] […] it's too late anyway” (502M, 50–59, early); the other said “whether they catch [LC] early or […] late, it doesn't really matter […] your longevity's marked regardless” (12M, 50–59, late). The woman attributed her delay in help-seeking to a number of factors, including it not being in her ‘nature’ to go to the doctor, initially finding common sense explanations for symptoms, and a belief that “lighting doesn't strike twice” (508 W, 50–59, late) which she described as denial that she might have LC so soon after her father had died of it. She did not express the opinion that consulting her GP would be futile, but described consulting a number of times over the course of a year before feeling so ill that she demanded further tests.

## 7 Discussion and conclusions

Despite concerns about men's help-seeking behaviours and (under-)use of health care having long been at the forefront of the men's health agenda (Galdas et al., 2005, Hunt et al., 2010), research has shown that the links between performances of gender and accounts of help-seeking for illness are far from simple and static. Attention is increasingly being drawn to ways in which expressions of masculinity in relation to help-seeking can vary as well as to similarities between men's and women's accounts of help-seeking (Farrimond, 2011, Galdas et al., 2007, Galdas et al., 2010, Johnson et al., 2012, O'Brien et al., 2005, Townsend et al., 2006, Townsend et al., 2008). The findings presented here add to this body of research, providing evidence of striking similarities in how men and women accounted for their interpretations of, and responses to, symptoms later diagnosed as LC. Both men's and women's narratives were heavily informed by culturally-embedded moral frameworks of responses to symptoms, and reconstructing their own moral integrity in their accounts was closely tied to the delicate balancing act of presenting the period leading up to consulting to their GP. Dual notions of ‘responsibility’ were evident across accounts, as men and women were keen to emphasise that they were stoic in response to symptoms and responsible health service users who did not ‘over-use’ the GP (see also, Llanwarne et al., 2017); they also portrayed themselves as having sought explanations for their symptoms and taken sensible, appropriate and timely actions in response. Participants who described symptoms which were less recognisable as LC (or easy to ascribe to ‘common sense’ explanations) emphasised that they had not suspected cancer, perhaps implying that they might have reacted differently if they had, and indicating a lack of knowledge among men and women as to the range of ways in which LC can become manifest. Participants who described symptoms more commonly associated with cancer were careful to highlight that they had not immediately assumed that cancer was the cause, perhaps as a means of defending their subsequent actions and avoiding being seen as irrational or over-reacting. While describing symptoms, participants were engaged in the task of presenting themselves as having interpreted these rationally, using their common sense, knowledge of their bodies, perceptions of risk associated with their lifestyle, and knowledge of the signs of cancer.

The moral balancing act involved in narrating this period of their illness experience also required participants to, on the one hand, show how they had not ignored symptoms of a serious illness by actively seeking out rational explanations, while, on the other hand, demonstrate that they had not panicked or over-reacted. It is highly significant, and contrary to common stereotypes, that both men's and women's narratives were equally influenced by these moral frameworks. Our analysis by gender provided no evidence that men went to greater efforts than women to present themselves as stoic or to justify their use of health services any more than women or that women were any more likely than men to present themselves as sensible and responsible in response to their symptoms nor to blame themselves more than men for any delay in help-seeking.

It is not surprising, given their diagnosis of LC, that men's and women's accounts appeared to be strongly influenced by moral frameworks (Chapple et al., 2004, Corner et al., 2005). However, given Galdas et al.'s call to go “beyond the masculine-feminine binary” (Galdas et al., 2010) of stereotypes that women are more likely to take care of their health but men align themselves with stoic or ‘don't care’ attitudes (Robertson, 2003, Robertson, 2006), it is significant that there were so few differences in how men and women presented their moral identities. Others have suggested that diagnosis with a serious illness can be seen, by men, as a legitimate reason to display identities and actions which align more with the expectations that they ‘should care’ (Robertson, 2003, Wenger, 2013). Indeed, in societies where maintaining health is viewed as a moral responsibility, both men and women may fear the consequent stigma of not responding to symptoms in culturally approved ways (Townsend et al., 2014, Wenger, 2013), and this may be heightened by a LC diagnosis.

Our findings also reinforce others' suggestions that becoming ill and recognising yourself as such can be complex and prolonged processes (Corner et al., 2005, Corner et al., 2006). For men and women, not recognising symptoms as something out of the ordinary, and efforts to manage them for as long as possible, are presented as causes of delay and barriers to help-seeking for LC (Corner et al., 2005, Corner et al., 2006, Smith et al., 2005). Indeed, research suggests that even when LC patients consult their GP, they often do not report symptoms which they class as being ‘normal’, such as a cough (Brindle et al., 2012). Although men and women may have emphasised the ways in which they normalised symptoms as a means of avoiding blame for any delay in help-seeking, Brindle et al. (2012, p10) reported that patients they interviewed also normalised symptoms which they experienced after their LC diagnosis, suggesting that “the association of episodic, non-specific symptoms with normal processes appears commonplace for those feeling well, even when LC provides a potential explanation for symptoms”.

It is necessary to highlight the limitations inherent in the retrospective nature of the data we have presented, particularly the potential for recall bias in participants' narration of events leading up to them consulting their GP, especially for participants who experienced symptoms over many months or years (Corner et al., 2005, Molassiotis, 2009). This would have been more of a limitation had we been analysing the data to reconstruct factual timelines, but is less so given the focus of our analysis was to illuminate participants' presentations of their interpretations of, and reactions to, symptoms (Townsend et al., 2014). Others have suggested that participants, with the benefit of hindsight, may retell their story attributing different significance to symptoms and downplaying personal culpability for delay (Corner et al., 2005, Molassiotis, 2009). We agree but see the existence of such features in participants' accounts as one of the strengths of this study. Because our analysis involved investigating the moral frameworks impacting participants’ presentations of themselves, we paid attention to evidence which suggested they were trying to portray themselves, or their interpretations and actions, as aligned (or not) with culturally-embedded moral ideals. For example, if participants appeared to be downplaying their role in delay, we explored the ways in which they were doing so, investigated to what ends this was being done, and compared any evidence by gender.

The findings we have outlined highlight the value of conducting gender comparative research with men and women with comparable underlying morbidity (Hunt et al., 2010), in this case diagnosis with a serious and potentially life-threatening cancer. To avoid the reinforcement of misleading essentialist assumptions, which have implications for men's and women's consulting behaviours and the care they receive (Lyratzopoulos et al., 2012, Schoenberg et al., 2003), the findings presented support the view that it is time to challenge binary gender stereotypes of ‘stoical’ men being more reluctant to consult when they ought and ‘anxious’ women being willing to consult with only minor or trivial symptoms, and recognise that both men and women need support and encouragement to consult their doctor for further investigation of significant or concerning bodily changes.
